# Efgartigimod as a salvage therapy for anti-NMDAR encephalitis patients after first-line treatment failure: a case series

**DOI:** 10.3389/fneur.2026.1842709

**Published:** 2026-08-14

**Authors:** Yanbo Zhang, Zhijiao Song, Lulu Zheng, Lantao Liang, Hui Zhao, Hui Bu

**Affiliations:** 1 Department of Neurology, The Second Hospital of Hebei Medical University, Shijiazhuang, Hebei, China; 2 Key Laboratory of Clinical Neurology, Ministry of Education, Hebei Medical University, Shijiazhuang, Hebei, China; 3 Neurological Laboratory of Hebei Province, Shijiazhuang, Hebei, China

**Keywords:** autoimmune encephalitis, efgartigimod, immunoglobulin G, neonatal fc-receptor, N-methyl-D-aspartate-receptor

## Abstract

**Background:**

As an Fc receptor inhibitor, efgartigimod (EFG) could reduce serum IgG level, which might have a positive effect in the treatment of anti-N-methyl-D-aspartate-receptor (NMDAR) encephalitis (NMDARE). This study aimed to evaluate the treatment effects and safety of EFG on NMDARE patients following a first-line treatment failure.

**Methods:**

We retrospectively reviewed five NMDARE patients who failed in first-line treatment. All patients received intravenous EFG (10 mg/kg/week). We conducted neurological function evaluation, cerebrospinal fluid (CSF) and hematological examination, magnetic resonance imaging (MRI), and electroencephalogram (EEG) in all patients to evaluate the treatment effects of EFG.

**Results:**

After EFG treatment, the mean modified Rankin Scale (mRS) score decreased from 4.00 ± 0.70 at baseline to 1.60 ± 0.89, and the mean Clinical Assessment Scale in Autoimmune Encephalitis (CASE) score decreased from 14.6 ± 6.58 to 2.80 ± 2.95. The average period from EFG treatment to significant nerve function recovery (defined as mRS reduction ≥2) was 2.40 ± 2.19 weeks. Moreover, following a week after EFG treatment, the average serum IgG level decreased from 16.13 ± 7.47 g/L at baseline to 7.75 ± 5.17 g/L. Additionally, at 4 weeks post-EFG treatment, the IgG level decreased to 4.97 ± 2.30 g/L. Meanwhile, the anti-NMDAR antibody in CSF turned negative after EFG treatment in four out of five patients. The EFG helped in alleviating the abnormalities identified by MRI or EEG evaluations.

**Conclusion:**

The EFG can rapidly improve neurological function and enhance prognosis in NMDARE patients after first-line treatment failure. Meanwhile, no serious adverse effects were identified. The EFG treatment mechanism might be associated with the elimination of IgG, which contains anti-NMDAR antibodies.

## 1 Introduction

Anti-NMDAR (N-methyl-D-aspartate-receptor) encephalitis (NMDARE) is the most common autoimmune encephalitis that shows fatal clinical symptoms and poor prognosis. Dalmau et al. reported that the estimated mortality for NMDARE was 4%, and the time from disease onset to the patient’s death was 3.5 months. Approximately 30% of the patients require intensive care unit admission. Moreover, >25% of NMDARE patients remained severely disabled post-treatment (1). Currently, there is no etiological treatment for NMDARE. Although the majority of NMDARE patients benefit from first-line immunotherapy that includes intravenous methylprednisolone pulse (IVMP) therapy, intravenous immunoglobulin (IVIG), and plasma exchange (PE), there were still 19–33% patients who did not respond well to the first- or second-line treatments. These patients face persistent neurological and psychological issues (2–5). Furthermore, using corticosteroids might cause infection, osteoporosis, and osteonecrosis. Since PE is an invasive procedure, it might be difficult to perform in pediatric patients and can cause virus transmission and coagulation disorders (6). IVIG may benefit patients with NMDARE by modulating humoral immunity, neutralizing pathogenic autoantibodies, and promoting IgG catabolism through FcRn saturation, thereby reducing the functional impact of anti-NMDAR antibodies (7). IVIG is expensive, dependent on plasma donations, increases the lysosomal degradation of normal IgG (8). Second-line immunotherapies, including cyclophosphamide and rituximab, are commonly used in patients with NMDARE who respond inadequately to first-line treatment (9). However, cyclophosphamide (10) has relatively poor cellular selectivity. As a cell-cycle nonspecific cytotoxic agent, it may affect both proliferating cells and quiescent cells in the G0 phase, contributing to serious adverse effects such as bone marrow suppression and gonadal toxicity. Rituximab (11) depletes CD20-positive B cells and thereby reduces subsequent antibody production. Nevertheless, it does not directly remove pre-existing circulating antibodies, and its effect on antibody-producing CD20-negative plasma cells is limited.

Since the combination of anti-NMDAR antibodies and NMDAR is crucial in the pathogenesis of NMDARE, anti-NMDAR antibody elimination might become a new therapy for managing NMDARE (12). A human IgG Fab fragment, efgartigimod (EFG), accelerates the destruction of IgG and reduces the pathogenic IgG as well as IgG immune complexes (13). This leads to rapid and specific clearance of serum IgG levels, especially IgG1, which contains the anti-NMDAR antibody (14). Since EFG has demonstrated satisfactory treatment effects in autoimmune diseases like myasthenia gravis, primary immune thrombocytopenia, bullous pemphigoid, neuromyelitis optica, and Guillain-Barré syndrome, it might be possible that EFG might have a potentially positive effect in the treatment of NMDARE (15–18).

Currently, very few studies are available on the usage of EFG for managing NMDARE. In a multi-center study, Liu et al. found the EFG had a treatment effect similar to other immunotherapies like IVIG and immunoadsorption with staphylococcal protein A column (SPA-IA) (19). However, they had used EFG with IVMP and other second-line immunotherapies. Hence, the results might not truly reflect the treatment effects of EFG. Several other studies have shown the positive effects of EFG on NMDARE (20–22). However, all these studies were case reports, which had a limited scientific impact. Thus, we included patients with NMDARE who had an inadequate response to first-line immunotherapy and subsequently received EFG as salvage treatment, given its potential to directly promote the clearance of circulating IgG antibodies and thereby provide a more rapid and direct therapeutic approach compared with rituximab or cyclophosphamide. This study aimed to investigate the treatment effects and safety of EFG on NMDARE patients, especially in those patients who failed in first-line treatment.

## 2 Materials and methods participants

Being a retrospective, single-center study, we included autoimmune encephalitis patients with the following inclusion criteria (23): (1) Those who had at least one of the following symptoms, including psychosis, memory deficit, speech disturbances, seizures, movement disorders, impaired consciousness, autonomic dysfunction, and central hypoventilation; (2) Patients whose serum or CSF contained anti-NMDAR antibody, detected by cell-based assay, and (3) Those who did not respond to first-line treatment and subsequently, received salvage EFG treatment. The first-line treatment included at least two following treatments, such as IVMP, IVIG, and PE, for >2 weeks. Failure of first-line immunotherapy was defined as the mRS score remaining at 4 or higher and/or no sustained improvement in the most prominent symptoms, assessed by two experienced neurologists after 2–4 weeks of the treatment initiation (24). The exclusion criteria were: those diagnosed with acute or chronic infections (e.g., pulmonary infection), patients with an allergic history to EFG and insufficient medical records or test results, and those lost to follow-up.

### 2.1 Treatment strategy

All patients received first-line treatment when initially diagnosed with NMDARE. Patients were given methylprednisolone (1,000 mg/day) and immunoglobulin (0.4 g/kg/day) for three and 5 days, respectively. Subsequently, patients received oral methylprednisolone. Methylprednisolone’s maintenance dose was 60 mg/day. After that, the dose was reduced by 5 mg each week, until it reached 20 mg/day. The methylprednisolone dose was reduced by 2.5 mg every 2 weeks. The entire treatment lasted 6 months. One patient (Case 1) additionally underwent three sessions of plasma exchange.

EFG was initiated at least 2 weeks after completion of the last first-line therapy as a salvage treatment. All patients received intravenous EFG (10 mg/kg/week). The EFG treatment was terminated if the patient’s mRS<2 points. During EFG treatment, all immunotherapies were stopped except oral methylprednisolone’s maintenance dose.

Other adjuvant treatments included anti-epilepsy drugs (sodium valproate, levetiracetam), antipsychotic drugs (olanzapine, clonazepam), and antibiotics. Their usage was determined by neurologists based on the patient’s symptoms.

### 2.2 Evaluations and examinations

The patients were assessed for neurological function evaluation, CSF assessment, hematological examination, MRI, and electroencephalogram, and the final prognosis. (1) The modified Rankin Scale (mRS) and Clinical Assessment Scale in Autoimmune Encephalitis (CASE) were used for neurological function assessments (25, 26). For each patient, two neurologists conducted independent assessments. They were blinded to efgartigimod treatment decisions, protocol, and timing. If results were not unanimous, the two neurologists’ consensus was used as the final score. Neurological function was evaluated weekly post-NMDARE diagnosis until stabilization (mRS and CASE unchanged across three evaluations). Subsequently, the neurological function evaluations were performed at 1, 3, and 6 months, as well as final follow-up. (2) CSF examination included complete white blood cell count, biochemical analyses, and anti-NMDAR antibody concentration. The CSF examinations were performed at the onset of NMDAR, before EFG treatment, and 4 weeks after EFG treatment. (3) Hematological examination also included complete blood cell count, biochemical analyses, and anti-NMDAR antibody concentration. The hematological and neurological examinations were performed every week. (4) The MRI sequences included T1, T2, FLAIR, DWI and enhanced scans. The evaluations of MRI images were conducted by two neurologists independently, and the quality control measures MRI images were similar to neurological function assessment. The patients received video-EEG exams as well. The time-points of MRI and EEG exams were the onset of NMDARE and 4 weeks after EFG. (5) The time from EFG treatment to neurological function stabilization (the mRS and CASE values were the same in three evaluations), the incidence and severity of permanent nerve dysfunction, recurrence (after the initial symptoms improved or stabilized for at least 2 months, new symptoms appeared or previous symptoms were aggravated, accompanied with enhanced CSF/serum anti-NMDAR antibody levels) and other clinical complications were consequently investigated during the follow-up period. These parameters were considered as the patient’s final prognosis (4).

### 2.3 Outcome

The primary outcome was neurological function (mRS and CASE) at 4 weeks after EFG treatment. Secondary outcomes included the time of neurological function improvement, the concentration of serum or CSF IgG and anti-NMDAR antibody, and the recovery on MRI and EEG results. The safety outcome of EFG included the adverse effects, infection, and other complications caused by EFG.

### 2.4 Statistical analysis

Statistical analyses were performed using SPSS, version 19.0 for Windows (IBM, Armonk, NY). Continuous and categorical variables were expressed as mean ± standard deviation and frequencies, respectively. Because of the extremely small sample size of only five patients, statistical inference would be inherently unstable and could give a misleading impression of robustness. Therefore, only descriptive analyses were performed in this study, including individual patient trajectories and effect sizes, rather than statistical significance testing.

## 3 Results

### 3.1 Demographic information, clinical features, and treatment procedure

A total of 26 NMDARE patients were initially reviewed. Among them, five patients (4 males and 1 female) failed in first-line treatment and were included. The average age was 36.60 ± 18.24 years (range from 16 to 62 years). None of the patients reported a previous history of tumors or autoimmune diseases. In 4/5 patients, infectious diseases were identified before the onset of NMDARE. In one patient, a hemorrhoid surgery was performed 4 weeks before NMDARE onset, which was considered a potential inducement. All patients underwent tumor screening, and no tumors were detected. In terms of clinical symptoms, epilepsy and cognitive impairment were observed in five and four patients, respectively. Other symptoms of central nervous dysfunction, like motor dysfunction, disturbance of consciousness, autonomic dysfunction, and brainstem dysfunction, were observed in 3/5 of the patients (Table 1).

**Table 1:** Demographic and clinical features of anti-NMDARE patients.

Case No.	Age at diagnosis (years)	Sex	Past medical history	History of prior infection	Smoking and alcohol consumption	Comorbidities	Symptoms
1	18	Male	No	No	Both negative	No	Epilepsy, Psychiatric disorders, Cognitive impairment, Motor dysfunction, Disturbance of consciousness, Autonomic dysfunction, Brainstem dysfunction
2	28	Male	Sinusitis	Yes	Both negative	No	Epilepsy, Psychiatric disorders, Disturbance of consciousness, Autonomic dysfunction, Brainstem dysfunction
3	49	Female	Hemorrhoid surgery	Yes	Both negative	No	Epilepsy, Psychiatric disorders, Cognitive impairment, Disturbance of consciousness, Autonomic dysfunction, Brainstem dysfunction
4	62	Male	No	Yes	Both negative	No	Epilepsy, Cognitive impairment
5	26	Male	No	Yes	Both positive	No	Epilepsy, Cognitive impairment

NMDAR, N-methyl-D-aspartate-receptor.

All five patients received first-line treatment before the EFG therapy. All patients received IVIG and IVMP simultaneously. Due to a serious nerve dysfunction, PE was performed in one patient (case 1). After that, patients received the maintenance dose of steroid. Subsequently, other first-line treatments were withdrawn. Adjuvant treatments, like anti-infection therapy, anti-epileptic drugs, and psychotropic medication, were used, based on each individual’s situation. When the patient was considered to have failed in first-line treatment, intravenous EFG (10 mg/kg/week) was administered as a salvage therapy. The average period from NMDARE onset to EFG application was 5.4 ± 2.3 weeks. Moreover, EFG therapy was sustained for 4 weeks in three out of five patients. In one patient, EFG therapy was continued for 7 weeks. In another patient, because an immediately nerve function recovery was identified, EFG was applied only once (Table 2).

**Table 2:** Treatment procedures for anti-NMDARE patients.

Case No.	First-line treatment	Time from first line treatment to EFG	EFG dose and time	Other treatment
1	IVIG+IVMP+PE	8 weeks	10 mg/kg/week, 7 weeks	Olanzapine, risperidone, valproate, levetiracetam, ceftriaxone
2	IVIG+IVMP	5 weeks	10 mg/kg/week, 4 weeks	Midazolam, valproate, levetiracetam, ceftriaxone
3	IVIG+IVMP	7 weeks	10 mg/kg/week, 1 weeks	Olanzapine, estazolam, valproate, levetiracetam, lamotrigine, ceftriaxone
4	IVIG+IVMP	2 weeks	10 mg/kg/week, 4 weeks	Valproate, levetiracetam
5	IVIG+IVMP	5 weeks	10 mg/kg/week, 4 weeks	Valproate, levetiracetam, ceftriaxone

EFG, efgartigimod; IVIG, intravenous immune globulin (0.4 g/kg/d, 5 days); IVMP, intravenous methylprednisolone (1,000 mg/day, 3 days); NMDAR, N-methyl-D-aspartate-receptor; PE, plasma exchange.

### 3.2 Neurological function evaluation

**Figure 1:**
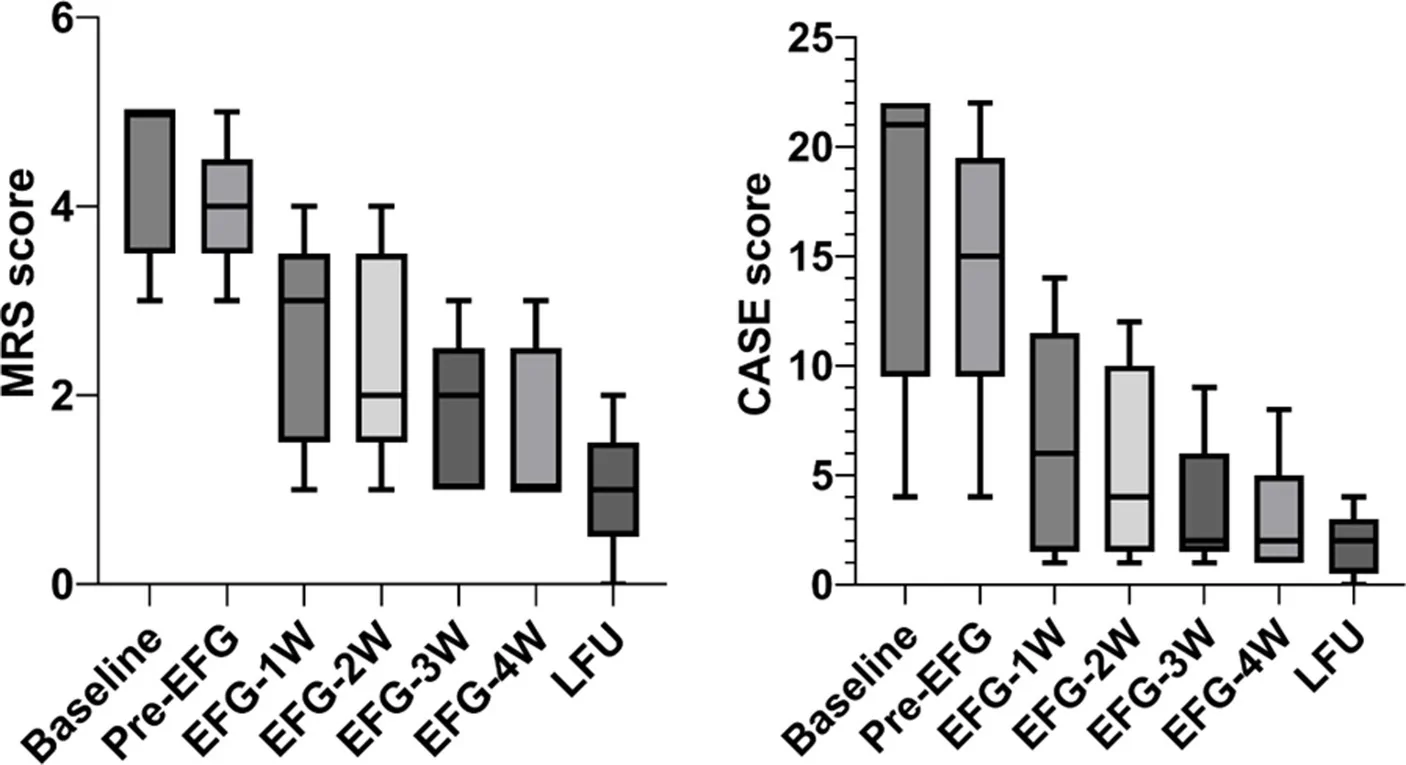
Changes in mRS and CASE values in five patients. The changes in CASE or mRS for five patients were monitored during hospitalization, before EFG administration, 1/2/3/4 weeks after EFG treatment, and at the last follow-up.

Significant neurological dysfunction was identified in all patients. The average mRS and CASE values at baseline were 4.40 ± 0.89 and 16.8 ± 7.73, respectively. After 2 weeks of first-line treatment, the average mRS and CASE values were 4.00 ± 0.70 and 14.6 ± 6.58, which demonstrated almost no reduction. EFG could obviously improve the nerve function, which was time dependent. At one week post-EFG treatment, the mRS and CASE values were reduced in 3/5 patients, with an average decrease of≥2 in CASE. At 4 weeks, the mean modified Rankin Scale (mRS) score decreased from 4.00 ± 0.70 at baseline to 1.60 ± 0.89, and the mean Clinical Assessment Scale in Autoimmune Encephalitis (CASE) score decreased from 14.6 ± 6.58 to 2.80 ± 2.95. Finally, all five patients achieved a significant nerve function recovery (mRS decrease ≥2). The average time from EFG treatment to significant nerve function recovery was 2.40 ± 2.19 weeks. The average mRS and CASE values were 1.00 ± 0.71 and 1.80 ± 1.48 at the final follow-up, with all patients’ mRS ≤ 2. One case (case 1) showed a complete nerve function recovery (mRS = 0, CASE = 0). The changes in nerve function scales suggested that EFG could reverse the nerve dysfunction in NMDARE and relieve the patients’ clinical symptoms (Figure 1).

### 3.3 Cerebrospinal fluid examination

The changes in CSF evaluation included elevated WBC counts (46.20 ± 54.87 ×10^6^/L) and protein concentrations (0.50 ± 0.22 g/L) in NMDARE patients. Other biochemical parameters like glucose and chloride remained at normal levels. In these five patients, first-line treatment did not influence the WBC count and biochemical parameters of CSF. The EFG reduced the WBC count (41.80 ± 48.01 vs. 7.60 ± 7.27) and protein concentration (0.51 ± 0.25 g/L vs. 0.26 ± 0.14 g/L) in CSF. Moreover, EFG significantly reduced the anti-NMDAR antibody titer. In 4/5 patients, the anti-NMDAR antibody turned negative post-EFG. In another patient, the anti-NMDAR antibody titer reduced from 1:100 to 1:10 (Table 3).

**Table 3:** Cerebrospinal fluid examination results of anti-NMDARE patients.

Time	Case No.	Pressure (mmH_2_O)	White blood cell count (x10^6^/L)	Lymphocyte, %	Glucose (mmol/L)	Chloride (mmol/L)	Total protein (g/L)	Anti-NMDAR antibody (tiler)
Baseline	1	80	4	100	3.02	123.1	0.21	1:100
	2	190	28	82	3.32	126.6	0.35	1:32
	3	80	53	88	3.14	120.7	0.54	1:10
	4	140	8	87	3.28	122	0.75	1:10
	5	225	138	65	2.58	124.8	0.66	1:32
	Average	143.00 ± 64.96	46.20 ± 54.87	84.40 ± 12.70	3.07 ± 0.30	123.44 ± 2.32	0.50 ± 0.22	-
Before EFG treatment	1	80	6	100	3.25	125.4	0.18	1:100
	2	180	24	89	3.14	124.1	0.39	1:10
	3	120	46	88	3.85	123.8	0.50	1:10
	4	150	10	93	3.22	120.5	0.83	1:10
	5	180	123	76	2.65	122.7	0.64	1:32
	Average	142.00 ± 42.66	41.80 ± 48.01	89.20 ± 8.76	3.22 ± 0.43	123.30 ± 1.84	0.51 ± 0.25	-
4 weeks after EFG treatment	1	100	1	100	4.09	125.1	0.04	1:10
	2	140	5	100	3.66	125.7	0.33	Negative
	3	110	7	100	3.93	116.8	0.38	Negative
	4	130	5	100	3.66	125.7	0.33	Negative
	5	140	20	100	3.44	126.2	0.24	Negative
	Average	124.00 ± 18.17	7.60 ± 7.27	100.0 ± 0.0	3.76 ± 0.26	123.90 ± 3.99	0.26 ± 0.14	-

EFG, efgartigimod; NMDAR, N-methyl-D-aspartate-receptor.

### 3.4 Hematological examination

The WBC count increased in two patients. One of them showed enhanced CRP levels. In other patients, hematological examination parameters demonstrated negative results, except for the anti-NMDAR antibody concentration. High level of anti-NMDAR antibody concentration (tiler≥1:100) were found in three patients, and moderate level of anti-NMDAR antibody concentration (tiler = 1:32) was found in one patient. In another patient, serum anti-NMDAR antibody demonstrated a negative result.

**Figure 2:**
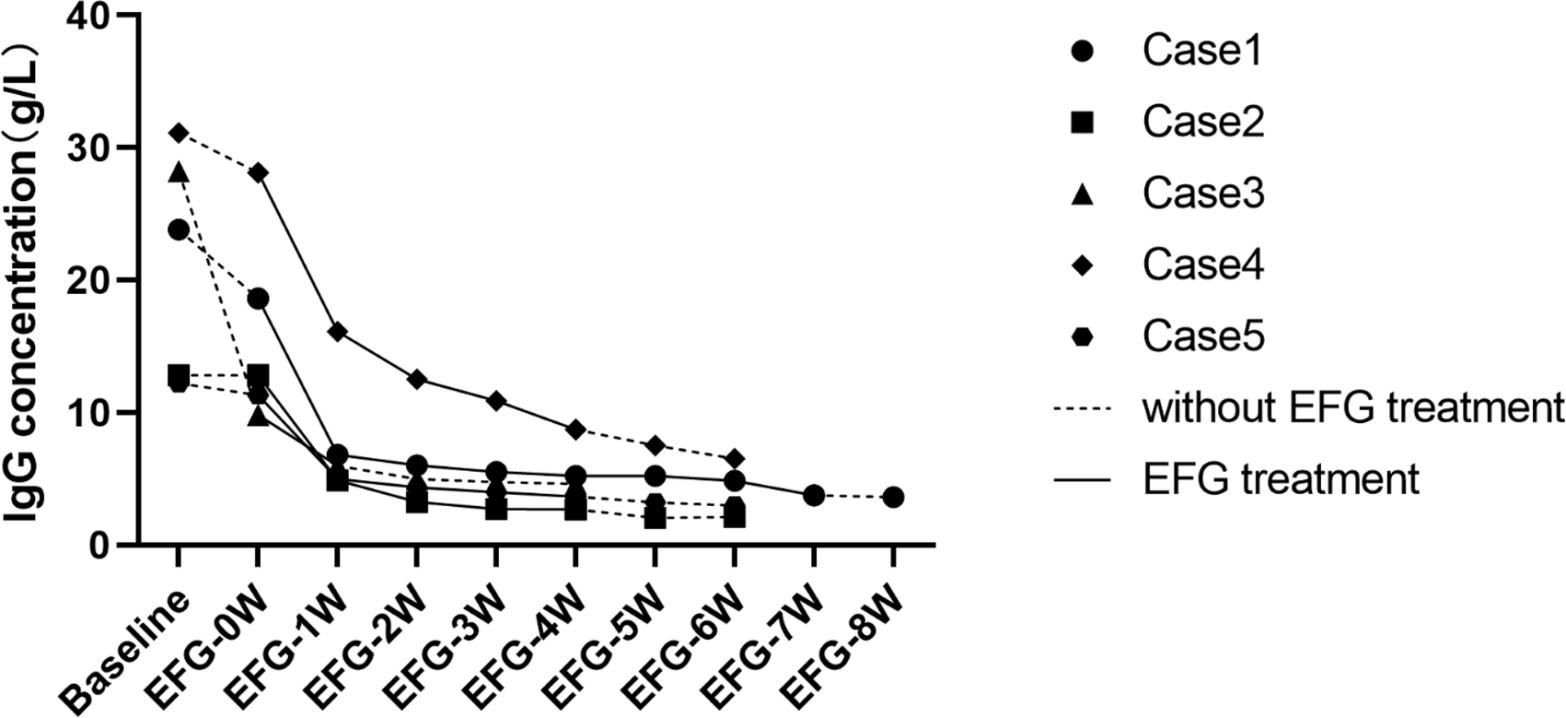
Individual trajectories of serum IgG levels in five NMDARE patients. Each symbol/line style represents one patient. Solid lines indicate the period of active EFG treatment; dashed lines indicate periods without EFG treatment (before initiation or after discontinuation).

The EFG treatment reduced the serum IgG levels. At one week after EFG treatment, the average serum IgG level decreased from 16.13 ± 7.47 g/L at baseline to 7.75 ± 5.17 g/L. The IgG level further decreased to 4.97 ± 2.30 g/L at 4 weeks after EFG treatment. Meanwhile, serum anti-NMDAR antibody detection results turned negative in three patients, and the anti-NMDAR antibody titer was significantly reduced in one patient. At final follow-up, all patients treated with EFG demonstrated negative results of the serum anti-NMDAR antibody test (Table 4; Figure 2).

**Table 4:** Hematological examination results of anti-NMDARE patients.

Time	Case No.	Cell count						Biochemical exams			Immunological exams	
		RBC (x10^12^/L)	WBC (x10^9^/L)	NE, %	LYM, %	M, %	PLT (x10^9^/L)	ALB (g/L)	GLB (g/L)	CRP (mg/L)	IgG (g/L)	Anti-NMDAR antibody (tiler)
Baseline	1	3.64	21.81	85.0	8.3	6.0	149	36.1	17.4	92.70	23.8	1:320
	2	3.51	5.56	53.2	19.9	14.8	352	34.0	12.8	7.24	12.8	1:100
	3	4.06	4.41	46.1	41.8	11.4	435	35.4	22.8	9.51	28.2	Negative
	4	4.29	6.30	81.7	16.0	2.2	250	38.1	41.8	5.60	31.1	1:100
	5	4.37	14.95	82.1	10.5	1.0	293	43.4	27.2	9.22	12.2	1:32
	Average	3.97 ± 0.38	10.61 ± 7.52	69.62 ± 18.45	19.30 ± 13.38	7.08 ± 5.92	295.8 ± 107.46	37.4 ± 3.67	24.4 ± 11.14	24.85 ± 37.96	21.62 ± 8.72	
Before EFG treatment	1	3.38	16.80	81.0	10.3	8.0	163	32.1	16.0	36.70	18.6	1:320
	2	3.69	6.58	76.1	16.9	6.6	334	36.0	12.3	4.38	12.8	1:100
	3	4.16	4.68	65.6	25.8	7.4	435	32.4	24.8	6.95	9.83	Negative
	4	4.04	6.14	76.9	18.0	4.2	263	39.1	33.8	4.98	28.1	1:100
	5	4.39	11.36	72.1	20.5	6.3	283	34.4	31.2	8.02	11.3	1:32
	Average	3.93 ± 0.399	9.11 ± 4.98	74.34 ± 5.82	18.3 ± 5.64	6.50 ± 1.45	295.6 ± 99.64	34.80 ± 2.88	23.62 ± 9.34	12.21 ± 13.78	16.13 ± 7.47	
4 weeks after EFG treatment	1	5.29	8.50	66.1	23.4	9.6	211	48.8	19.7	11.70	5.21	1:100
	2	4.29	9.21	69.3	10.0	5.4	267	36.6	12.4	17.03	2.67	Negative
	3	3.98	4.02	51.4	37.0	10.0	376	32.7	19.4	7.50	4.62	Negative
	4	3.93	5.40	87.7	10.4	1.8	165	41.2	20.6	6.40	8.7	Negative
	5	4.58	6.21	65.6	24.8	8.4	259	36.6	20.8	5.90	3.65	Negative
	Average	4.41 ± 0.55	6.67 ± 2.16	68.02 ± 12.99	21.12 ± 11.29	7.04 ± 3.44	255.6 ± 78.80	39.18 ± 6.16	18.58 ± 3.50	9.71 ± 4.69	4.97 ± 2.30	

ALB, albumin; CRP, C-reactive protein; EFG, efgartigimod; GLB, globulin; LYM, lymphocyte; M, monocyte; NE, neutrophils; NMDAR, N-methyl-D-aspartate-receptor; PLT, platelet; RBC, red blood cell; WBC, white blood cell.

### 3.5 MRI and electroencephalogram

**Figure 3:**
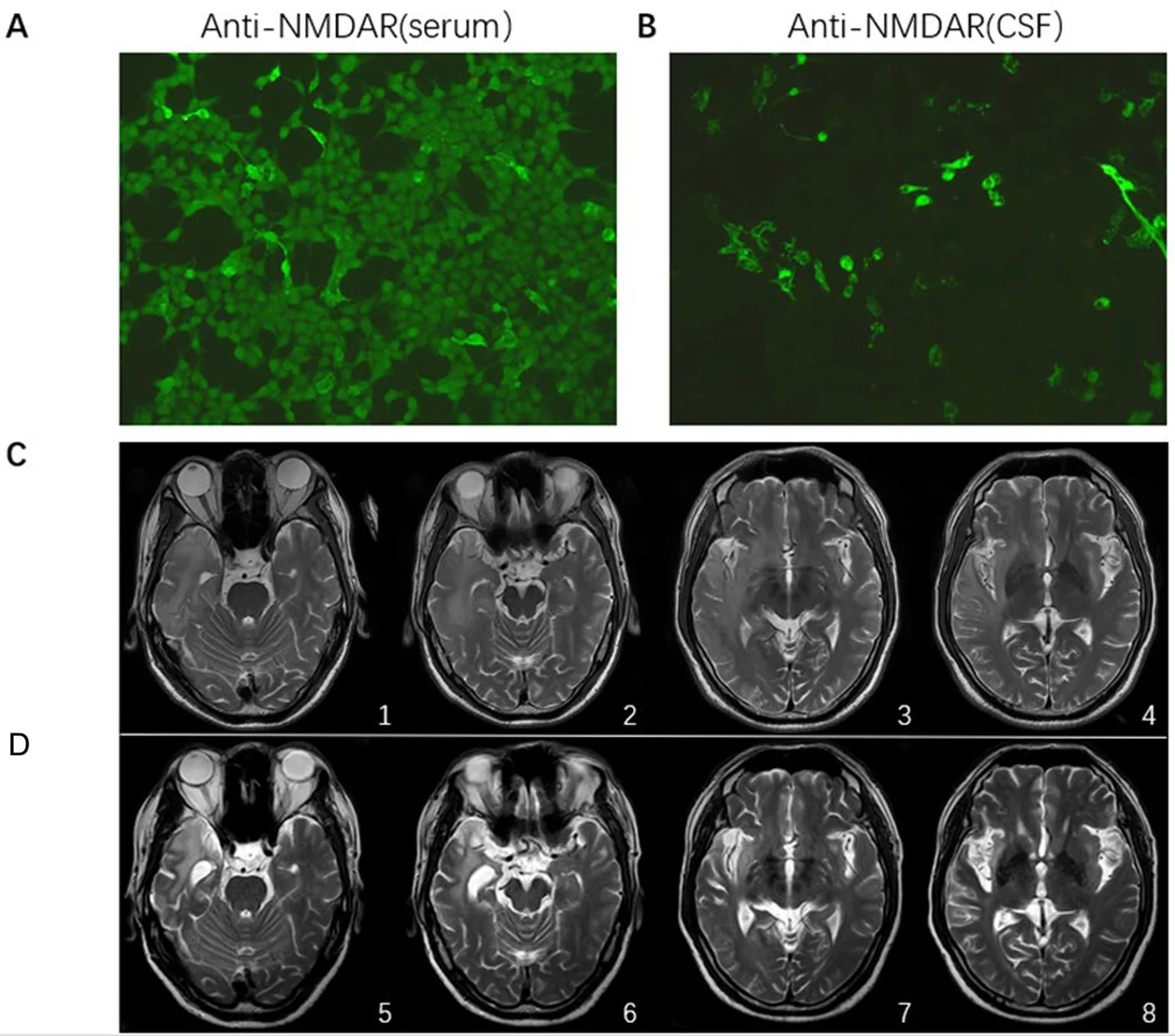
NMDAR antibody and cranial MRI changes of patient no. 4 **(A,B)** show serum and CSF positivity for NMDAR antibody. **(C)** 1–4 Show lesions with abnormal signals in the right frontoparietal lobe, bilateral temporal insulae, and right thalamus on FLAIR sequences before EFG treatment. **(D)** 5–8 Display reduction in lesion size and concomitant alleviation of perilesional edema post-EFG. FLAIR, fluid-attenuated inversion recovery; NMDAR, N-methyl-D-aspartate-receptor.

In four out of five patients, signs of encephalitis were identified on MRI images at baseline treatment. The signal abnormalities were primarily found in the cortex of the frontal, temporal, parietal, and insular lobes, respectively. In one patient, focal leptomeningeal enhancement was observed in the left parietotemporal region. In another patient, no abnormality was found on the MRI images. In all patients with MRI abnormalities, the lesions observed on MRI images significantly reduced after EFG treatment. The MRI results were negative in two patients. The area of the lesion reduced, and the brain tissue swelling was relieved in the other two patients (Table 5). The MRI images in a typical case are shown in Figure 3.

**Table 5:** MRI and electroencephalogram results of anti-NMDARE patients.

Time	Case No.	MRI findings	Electroencephalogram findings
Baseline	1	Cortical signal abnormalities in bilateral frontal & temporal lobes, left parietal & insular cortex	Diffuse background theta activity
	2	No abnormality detected	Diffuse background delta activity
	3	Multiple abnormal signals in bilateral frontoparietal lobes & corona radiata.	Diffuse delta background with multifocal spikes/sharp waves
	4	Abnormal signals in the right frontoparietal lobe & bilateral temporal insulae & right thalamus	No abnormality detected
	5	Focal leptomeningeal enhancement in the left parietotemporal region	Predominant delta activity; mild slowing of the occipital rhythm
4 weeks after EFG treatment	1	No abnormality detected	No abnormality detected
	2	No abnormality detected	scattered theta waves in the background activity
	3	reduction in lesion size and concomitant alleviation of perilesional edema	No abnormality detected
	4	reduction in lesion size and concomitant alleviation of perilesional edema	No abnormality detected
	5	No abnormality detected	scattered theta waves in the background activity

EFG, efgartigimod; MRI, magnetic resonance imaging; NMDAR, N-methyl-D-aspartate-receptor.

In terms of EEG findings, four out of five patients showed severe to moderate abnormalities, including extensive theta activity in one patient (severe abnormality), diffuse background theta activity in two patients (moderate abnormality), and diffuse delta background with multifocal spikes/sharp waves in one patient (moderate abnormality). After EFG treatment, two patients’ EEG results were normal. In another two patients, the EEG showed mild abnormalities (occasional delta activity). However, none of the patients showed spikes/sharp waves (Table 5).

### 3.6 Prognosis

None of the patients showed recurrence at the final follow-up. Except for one patient who completely recovered, the other four patients reported permanent neurological dysfunction (including mental disturbance, dysmnesia, and dyssomnia) without the presence of other symptoms like epilepsy, motor dysfunction, autonomic dysfunction, and brainstem dysfunction. The mRS scores were 2, 1, and 0 in one, three, and one patients, respectively. The CASE scores were 4, 2, 1, and 0 in one, two, one, and one patient, respectively. The average mRS and CASE values were 1 ± 0.63 and 1.8 ± 1.33, respectively. The average time from EFG treatment initiation to neurological function stabilization was 5.8 weeks (Figure 1).

### 3.7 Adverse effects

A total of 6 adverse events (AEs) were recorded during the study period (Table 6), all of which were grade 1 or 2 according to CTCAE v5.0, with no grade ≥3 AEs or serious adverse events reported. Three AEs were considered related to efgartigimod: one grade 2 pulmonary infection (possible), one grade 1 hepatic impairment characterized by transient ALT/AST elevations that resolved spontaneously without intervention (probable), and one grade 1 allergic reaction (possible). The remaining three AEs were considered unrelated to efgartigimod, including one grade 1 and one grade 2 pulmonary infection, as well as one grade 1 urinary tract infection, all of which were pre-existing at baseline and did not progress following treatment.

**Table 6:** The potential adverse effects of EFG in anti-NMDARE patients.

Case No.	Any adverse event	Elevated level of hepatic enzymes	Pulmonary infection	Urinary tract infection	Allergic reaction
1	Yes	No	Yes	No	No
2	Yes	Yes	Yes	No	Yes
3	Yes	No	Yes	Yes	No
4	No	No	No	No	No
5	No	No	No	No	No

EFG, efgartigimod; NMDAR, N-methyl-D-aspartate-receptor.

## 4 Discussion

We obtained several significant findings. First, EFG exerted positive therapeutic effects in patients who responded poorly to first-line treatment, even without use of other second-line immunosuppressive agents. Second, the mechanism of EFG may be associated with the clearance of IgG and anti-NMDAR antibody levels. In these NMDARE patients, symptom relief (mRS and CASE reductions) was identified, accompanied by reduced IgG and anti-NMDAR antibody concentrations in either serum or CSF. In patients with higher anti-NMDAR antibody titers or higher IgG levels, the neurological dysfunctions were more serious and sustained for longer periods, and finally left compromised prognosis. Apart from clinical symptoms and laboratory test results, the abnormal changes in MRI and EEG evaluations might be restored by EFG. Third, the onset time of EFG was individualized; however, most of the patients achieved relief in NMDARE symptoms after 4 weeks of treatment. Although the appropriate dose for NMDARE treatment needs additional exploration, using EFG at a dose of 10 mg/kg/week was considered efficient and safe for most of them. In the future, EFG may be considered as a potential adjunctive therapy in NMDARE, particularly in combination with established first- or second-line immunotherapies. By rapidly reducing circulating NMDAR antibody levels, EFG may contribute to early symptom control and provide a therapeutic window for etiological treatment or other immunosuppressive therapies to take effect.

Previously, the EFG showed a positive effect in acute NMDARE patients. In a multi-center study by Liu et al., NMDARE patients received EFG at a dose of 20 mg/kg on the first and fifth day, respectively (19). At discharge, the average mRS decreased from 3.5 to 2.0, following a mean hospital stay of 23 days. Subsequently, the average mRS decreased further, reaching 1 and 0 points at one-month and three-month follow-up, respectively. Therefore, Liu et al. concluded that EFG can improve the symptoms of NMDARE patients during acute attacks. Two major differences distinguish our study from the study of Liu et al. First, in that study, patients with acute, untreated NMDARE were included. However, we considered those patients who failed to first-line treatment. Second, in the study by Liu et al., the EFG was used along with IVIG and other second-line immunotherapies; while, in our study, EFG was administered only in combination with maintenance-dose corticosteroids, without the use of other second-line immunosuppressive agents. Although maintenance-dose corticosteroids may still exert anti-inflammatory and immunosuppressive effects, they were continued mainly as part of a tapering strategy after prolonged high-dose corticosteroid therapy, aiming to reduce the risk of inflammatory rebound and adrenal insufficiency. Given the inadequate response to the preceding high-dose corticosteroid therapy, the clinical improvement observed after EFG initiation suggests that EFG may have contributed substantially to neurological recovery. In addition, in our study, similar neurological function recovery was identified after EFG treatment, but most (4/5) of the patients displayed persistent neurological dysfunction, instead of a complete recovery, which was observed in the study by Liu et al. This inconsistency may be due to the patient heterogeneity (primary NMDARE vs. refractory NMDARE) between the two studies. Meanwhile, the combination of multiple immune therapies might also influence the prognosis of NMDARE patients. Liu et al. also suggested that EFG can lower the serum pathogenic IgG autoantibody titers, which is superior to that of IVIG and comparable to SPA-IA. Although our study did not directly compare EFG with PE, these two approaches differ fundamentally in their mechanisms: PE physically removes circulating antibodies and other plasma constituents through extracorporeal circulation, whereas EFG selectively accelerates IgG degradation by blocking FcRn-mediated IgG recycling.

The appropriate dosage of EFG in NMDARE treatment is ambiguous. In the study by Dalakas et al., EFG was used to treat neurological autoimmune diseases, like myasthenia gravis, polymyositis, and neuromyelitis optica spectrum disorder (27, 28). The dose was 10 mg/kg, at four infusions per cycle (one infusion per week). Subsequently, the regimen was adjusted based on symptom improvement. Xu et al. reported a case of NMDARE treated with EFG. Although the patient received IVIG and IVMP, no obvious symptom improvement was observed. At 10 weeks after the initial diagnosis, the patient received EFG (800 mg). An additional dose of EFG (400 mg) given at one and 5 weeks caused a rapid neurological recovery. In the study by Liu et al., EFG (20 mg/kg) was administered on the first and fifth days post-diagnosis. To sum up, the appropriate dosage of EFG in NMDARE treatment needs additional exploration. However, administering EFG every week at a dose of 10 mg/kg to 20 mg/kg might achieve a significant clinical improvement. Notably, the rapid improvement observed in Case 3 after a single EFG infusion may be partly explained by her lower baseline serum anti-NMDAR antibody burden and individual disease dynamics, although the small sample size precludes any definitive conclusion.

As an Fc receptor inhibitor, EFG selectively reduces serum IgG concentrations. Theoretically, it also accelerates the exogenous IgG clearance (29). Therefore, an antagonistic effect might exist between EFG and IVIG, while the latter was a crucial first-line treatment of NMDARE. Based on this, the combination of IVIG and EFG is not recommended. However, some NMDARE patients had already received IVIG and failed to respond. It is still unclear if the EFG dose should be adjusted in this unique circumstance. In this study, serum IgG levels were monitored and were found to decrease after EFG administration, suggesting that the therapeutic effect of EFG in NMDARE may be associated with the reduction of pathogenic IgG, potentially including NMDAR antibodies. In addition, serum IgG levels may serve as an adjunctive and relatively objective indicator to guide EFG dose adjustment. Compared with clinical scales alone, serum IgG levels are less susceptible to subjective bias and may therefore help determine the timing of EFG discontinuation. However, further studies are still needed to validate the utility of serum IgG levels in guiding EFG dose adjustment and treatment discontinuation.

Our study had several limitations. Due to a low incidence, the number of NMDARE patients, especially those who failed first-line treatment, is very rare. Thus, our sample size was limited. In addition, the duration of final follow-up varied among patients, which may have introduced heterogeneity in the assessment of long-term neurological outcomes and recurrence. As a retrospective design without a control group, the impact of the result and conclusion was inconsiderable. Particularly, there was a lack of comparison between EFG and treatment with a similar mechanism, like IVIG and PE. Moreover, because all patients had previously received first-line treatment, delayed responses to first-line therapy, as well as the self-limiting natural course of the disease, might have been misattributed to the therapeutic effects of EFG. The adjuvant treatment and maintenance-dose of corticosteroid might relieve the symptoms of NMDARE, and could become another confounding factor in this study.

## 5 Conclusion

For NMDARE patients who failed first-line treatment, using EFG could achieve a sufficient treatment effect. The EFG can rapidly improve the neurological function and final prognosis in NMDARE patients. Further, no serious adverse effects were identified. The treatment mechanism of EFG might be associated with IgG clearance, which contains an anti-NMDAR antibody. Additional studies with a large sample size and prospective design are needed, which might focus on using EFG (or in combination with other first- or second-line therapies) as an alternative drug to IVIG or PE for treating NMDARE.

## Data Availability

The datasets presented in this article are not readily available because of ethical and privacy restrictions. Requests to access the datasets should be directed to the corresponding author.
